# Age of First Suicide Attempt in Men and Women: An Admixture Analysis

**DOI:** 10.1100/2012/825189

**Published:** 2012-05-02

**Authors:** Hilario Blasco-Fontecilla, Analucia A. Alegria, David Delgado-Gomez, Teresa Legido-Gil, Jeronimo Saiz-Ruiz, Maria A. Oquendo, Enrique Baca-Garcia

**Affiliations:** ^1^Department of Psychiatry, Fundacion Jimenez Diaz, IIS, CIBERSAM, Autonoma University, 28040 Madrid, Spain; ^2^Department of Psychiatry, University Hospital Ramón y Cajal, IRYCIS, CIBERSAM, 28049 Madrid, Spain; ^3^Department of Psychiatry, New York State Psychiatric Institute, New York, NY 10032, USA; ^4^Department of Signal Theory and Communications, Carlos III University, 28903 Getafe, Spain

## Abstract

*Objectives*. To define different subgroups of suicide attempters according to age at onset of suicide attempts. *Methods*. Participants were 229 suicide attempters (147 females; 82 males) admitted to a general hospital in Madrid, Spain. We used admixture analysis to determine the best-fitting model for the age at onset of suicide attempts separated by sex. *Results*. The best fitted model for the age at onset of suicide attempts was a mixture of two gaussian distributions. Females showed an earlier age at onset of suicide attempts in both Gaussian distributions (mean ± S.D.) (26.98 ± 5.69 and 47.98 ± 14.13) than males (32.77 ± 8.11 and 61.31 ± 14.61). Early-onset female attempters were more likely to show borderline personality disorder than late-onset female attempters (OR = 11.11; 95% CI = 2.43–50.0). *Conclusions*. Age at onset of suicide attempts characterizes different subpopulations of suicide attempters.

## 1. Introduction

 Suicide attempts (SA) represent a major public health problem with a lifetime prevalence ranging between 1.1% [1] and 5.9% [2]. Suicidal behavior (death and attempts) is frequently a complication of psychiatric diagnoses [3]. However, there is increasing evidence that suicidal behavior exists independently from major psychiatric disorders [4–6]. Indeed, it has recently been suggested that suicidal behavior should be considered a separate diagnostic category apart from major psychiatric conditions [7]. Because a SA is one of the most compelling predictors of completed suicide, an optimal categorization of suicide attempters may serve to improve current suicide prevention and intervention policies [8, 9].

 Some studies have documented that younger age at first SA is strongly associated with higher rates of family history of suicidal behavior [10] and childhood risk factors [11, 12]. Also, life events preceding SA have been found to vary depending on the age of the first SA. A study by Heikkinen et al. [13] found physical-and social-related problems becoming more prominent and employment and financial problems less prominent with increasing age. Age at onset (AAO) of SA may aid in delineating disorder subtypes of suicidal behavior [10, 12, 13] and diminish heterogeneity [14]. The definition of clear-cut phenotypes is critical to establish the genetic underpinnings of complex behaviors such as suicidal behavior [15, 16]. Precise delineation of subtypes of SA will facilitate future research on the course, family transmission, pathophysiology, and treatment responsiveness of suicide attempters. In addition, an adequate understanding of AAO is essential for implementing prevention of psychiatric disorders, including suicidal behaviors [17]. To date, only one threshold to differentiate subgroups according to the AAO in SA has been proposed. Recently, Slama et al. [18] found that the theoretical model that best explained the distribution of age at first SA was a mixture of two Gaussian distributions with a cut-off point of 26 years old. Those in the younger group were found to be more likely to have anxiety disorders, cannabis misuse, and a history of childhood emotional and physical abuse, but less likely to present major depressive disorder.

 Sex, as well as age, has been widely recognized as a sociodemographic correlate significantly associated with SA [19]. Despite obvious differences in prevalence, rates of psychiatric disorders, lethality of attempts [20], methods used [21], rates of risk factors for SA [22], and more importantly differences in AAO between men and women [9, 23, 24], previous research aiming to validate a threshold according to the age of first SA did not include analyses separately by sex [18]. We sought to fill the knowledge gap by defining different subgroups according to onset of SA in a sample of adult first SA men and women using admixture analysis. We also sought to examine differences in sociodemographic characteristics, psychiatric comorbidity patterns, risk factors for psychopathology, and clinical suicidal correlates at the time of first SA between onset subgroups by sex.

## 2. Subjects and Methods

### 2.1. Sample

 We assessed 229 suicide attempters (147 women and 82 men) with no previous history of SA admitted to two general hospitals in Madrid, Spain between 1999 and 2003, which provide free medical care to a catchment area of approximately 900,000 people. A SA was defined as a self-destructive behavior with the intention of ending one's life [25, 26]. Approximately 84% of approached suicide attempters consented to take part in our study. Suicide attempters who rejected study participation did not significantly differ in demographics from suicide attempter participants. After a complete description of the study, subjects provided written informed consent before participating in the study. The appropriate ethics committee approved the study.

### 2.2. Axis I and Axis II Psychiatric Disorders

 The Mini International Neuropsychiatric Interview (MINI) was used to establish DSM-IV Axis I diagnoses. The MINI is a short and efficient structured diagnostic interview to assess psychiatric disorders [27]. Axis II personality disorder diagnoses (PD) were assessed according to DSM-IV version of the International Personality Disorder Examination Screening Questionnaire (IPDE-SQ). This instrument was selected because of its validity, reliability, brevity, and the brief training needed for its use [28]. In order to increase specificity, we used adjusted cut-off points for each PD. Our approach generated criteria for diagnosis at a more stringent level. For example, to diagnose schizotypal PD, 7 out of 9 items were required instead of the 5 screening items suggested by the authors of the IPDE-SQ [29]. Further information on this strategy can be found elsewhere [30–32].

### 2.3. Risk Factors for Suicidal Behavior

 In order to examine the differential effect of known risk factors for SA in men and women, we grouped these variables into childhood adverse events, familial risk factors, and recent life events. Recent life events were assessed with the St. Paul Ramsey scale. This scale assesses the severity, from 1 (none) to 7 (catastrophic), of each life event the individual has experienced in the last month previous to the interview by category (i.e., marital, other interpersonal, and work-related) [33]. For this study, only those life events with ratings of severity of 4 and over were considered.

### 2.4. Additional Measures

 Correlates of the first suicidal attempt were assessed with the Weisman and Worden Risk Rescue Rating Scale (RRRS) [34] and the Suicide Intent Scale (SIS) [35]. The RRRS is a ten-item scale (scored 1 to 3) that evaluates the lethality of a suicide attempt. Lethality is the result of risk factors (method used, impaired consciousness, toxicity, reversibility, and treatment required) and rescue factors (location, person initiating rescue, probability of discovery, accessibility to rescue, and delay until discovery) [36]. Based on our previous clinical experience, we deliberately selected some items of the RRRS in this study (see Table 3). The SIS, a 15-item scale with good-to-excellent interrater reliability (0.81≤ intra-class correlation coefficient ≤0.95), was used to assess suicide intent. Each item is scored 0–2 giving a total score of 0–30.

 Additional measures of lifetime aggression and high impulsivity were assessed with the Aggression History Scale Revised (Brown-Goldwin Scale, BGS) [37] and the Spanish version of the Barratt Impulsivity Scale, version 11 (BIS-11) [38], respectively. The BGS consists of 11 items and assesses the frequency of aggressive behavior. Interrater reliability was found to be very high (*r* > .98) [37]. The BIS-11 has been extensively used in the study of impulsivity both in suicidal and nonsuicidal samples [39]. The BIS-11 contains 30 self-report items scored 0–4 (range of total score 0–120) divided into 3 subscales. Consistent with our previous studies, the BGS cut points for high values were 10.5 and 9.5 and the BIS cut-points were 50.5 and 46.5 for males and females, respectively, [39, 40].

### 2.5. Statistical Analysis

 We used admixture analysis to determine the number of subgroups according to the AAO in SA and the age at cut point in first SA men and women separately. The posterior probabilities of each group for each AAO in SA were computed given Bayes formula, which allowed us to determine the most probable subgroup for each patient. Subsequently, suicide attempters were categorized according to their maximum probability of belonging to each theoretical subgroup [19].

 Percentages were computed to derive sociodemographic, psychiatric diagnoses, risk factors, and SA and treatment correlates of AAO. A set of univariate logistic regressions analyses yielded odds ratios (ORs) and 95% confidence intervals (CI) indicating measures of association between the onset-groups (early- and late-onset) of SA and variables assessed in the study. We consider an OR statistically significant when its CI does not include 1. Analyses were conducted using Matlab and SPSS v16.0.

## 3. Results

**Figure 1:**
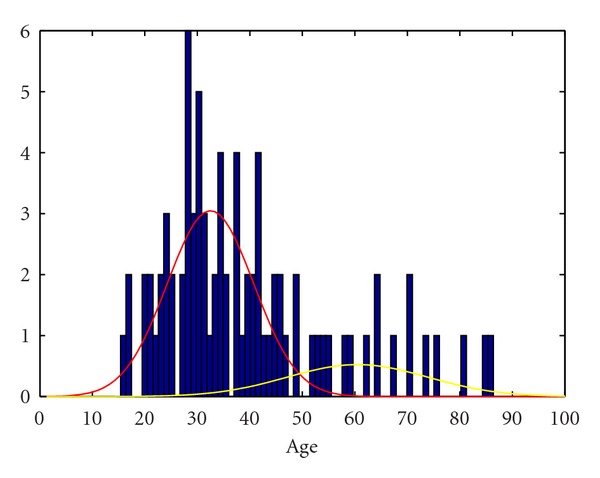
Theoretical distributions of ages at first SA in younger and older subgroups among men.

**Figure 2:**
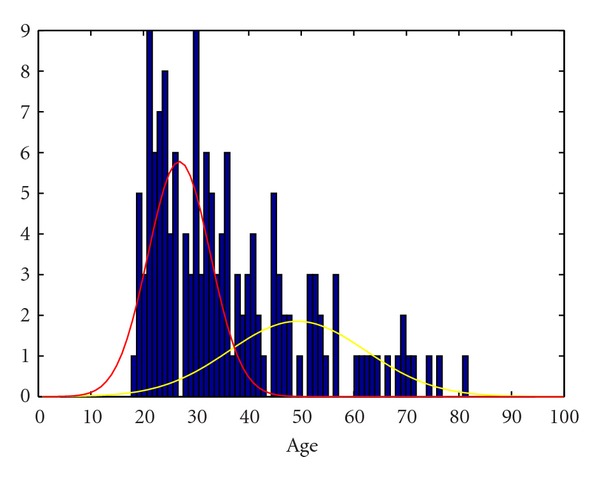
Theoretical distributions of ages at first SA among younger and older subgroups among women.

 When the whole sample's (*n* = 229) age at first SA was considered, the best-fitting model was a mixture of two Gaussian distributions with a cutoff point at 42 years of age. The early-onset subgroup had a mean age of 29.13 (SD = 6.87) years while the late-onset subgroup had a mean age of 51.44 (SD = 15.13) years. When data were analyzed separately by sex, the age at cut point of the Gaussian distributions and the mean age of the early-onset subgroup as well as the late-onset subgroup differed significantly between men and women. Among men (*n* = 82), the cutoff point was at 52 years of age, where the early-onset subgroup had a mean age of 32.77 (SD = 8.11) and the late-onset subgroup had a mean age of 61.31 (SD = 14.61) years. For women (*n* = 147), the age at cut-off point was 37 years of age. The early-onset female attempters had a mean age of 26.98 (SD = 5.69) while the late-onset subgroup of women had a mean age of 47.98 (SD = 14.13). See Figures 1 and 2. 

### 3.1. Women

#### 3.1.1. Sociodemographic Characteristics

 Sociodemographic characteristics of women and men with early and late onset of SA are depicted in Table 1. Older female attempters were less likely to be single and had higher odds of having 8 or less years of education, being born in a rural area and having religious beliefs compared to women in the younger group.

#### 3.1.2. Risk Factors and Stressful Life Events

 Risk factors and stressful events in the last month are shown in Table 2. Late-onset female attempters were more likely to report financial problems than those in the early-onset subgroup.

#### 3.1.3. Psychopathological Profile

Late-onset female attempters were over 3 times more likely to have a current diagnosis of dysthymia (OR = 3.86; 95% CI = 1.36–10.99) than the early-onset attempters. Additionally, they were significantly less likely to meet any anxiety disorder (OR = 0.42; 95% CI = 0.20–0.89), any eating disorder (OR = 0.21; 95% CI = 0.05–0.95), and any PD (OR = 0.37; 95% CI = 0.18–0.75), particularly borderline (OR = 0.09; 95% CI = 0.02–0.41) and dependent (OR = 0.07; 95% CI = 0.01–0.50) PD than their early-onset counterparts.

#### 3.1.4. Suicide-Related Measures and Their Correlates

 Suicide and clinical correlates of age at first SA by sex are shown in Table 3. SIS mean score was significantly higher among late-onset female attempters than among the early-onset subgroup. No differences were observed in the methods used, lethality, in the odds of being discovered, and in the intention of repetition between the early- and late-onset subgroups.

 Regarding clinical correlates of age at first SA, females in the late-onset group were significantly less likely to have high levels of impulsivity and aggression (see Table 3).

### 3.2. Men

#### 3.2.1. Sociodemographic Characteristics

 Late-onset male attempters have higher odds of being born in a rural area and to present disability than their early-onset counterparts (see Table 1). 

#### 3.2.2. Risk Factors and Stressful Life Events

 Similar patterns of childhood adverse events, familial risk for suicidal behavior and stressful life events in last month were observed between those in the early- and late-onset subgroups. The most common past month stressful life events in the early- and late-onset groups were problems related to the primary social support group (73.4%; 58.8%, resp.) and related to work (40.6%; 35.3%, resp.). 

#### 3.2.3. Psychopathological Profile

Our study showed no differences in the pattern of psychiatric diagnoses met between the early- and late-onset male subgroups. 

#### 3.2.4. Suicide-Related Measures and Their Correlates

 Late-onset male attempters were more likely to be discovered soon after attempting suicide than the early-onset group. Moreover, the late-onset group was less likely to have high levels of aggression than the younger male attempters. See Table 3. 

## 4. Discussion

 The theoretical function best fitting the distribution of mean AAO of SA was a mixture of two Gaussian distributions with a cut-off point of 42 years old. Slama et al. [18] reported a 26 years old cut-off point for their sample of suicide attempters. The difference in the age cut-off reported in our study is likely to be accounted by differences in the sample characteristics. In the study conducted by Slama et al. the sample consisted on a combination of first and recurrent SA aged as young as 10, whereas for the present study only first SA aged 18 and older were considered. The mean age of the early- and late-onset subgroups were significantly higher among male attempters than among females, which is consistent with some [9, 23, 24, 41] but not all [42, 43] studies. A number of reasons such as hormonal factors [44] and timing of puberty or sex-related stress factors [23] have been proposed to explain this time lag. Additionally, our results showed that, among females, those in the early- and late-onset subgroup differ in educational level, nativity area, psychiatric profile, levels of impulsivity, and aggression and intent of the SA. Early- and late-onset male attempters showed significant differences in nativity area, employment status, and aggression levels. 

### 4.1. Women

#### 4.1.1. Risk Factors and Stressful Life Events

 When we investigated the prevalence of childhood adverse events and familial risk factors for suicidality, we found similar patterns among females in the early- and late-onset subgroups. Moreover, the overall patterns of stressful live events in the previous month were also similar with the exception that those in the late-onset subgroup were more likely to report financial problems. In accordance with previous research [45], the majority of suicide attempters of both sexes presented with one or more stressful life events, particularly problems related to their primary support group. Haw and Hawton [46] reported that 80.6% of their sample of deliberate self-harm persons reported multiple life problems, the most frequent being troubles with spouse or partner. Overall, the similar rates of risk factors for suicidal behavior in both early- and late-onset subgroups observed in the current study suggest that childhood risk factors, familial history of suicidal behavior, and stressful live events in the previous month (except for financial problems) represent a similar risk for suicidal behavior in women regardless of age. 

 In our study, financial strain was the only stressful life event that differentiated between early- and late-onset female attempters. It is possible that the characteristics of the older female attempters in our sample may render them more vulnerable to financial strain. Financial stress has been associated with suicide at 50 years of age and older [47] and with the use of violent methods in both sexes [48]. 

#### 4.1.2. Psychopathological Profile

 Our study found that among females, those in the late-onset subgroup were more likely than their early-onset counterparts to have a current diagnosis of dysthymia, a disorder that increases the risk for suicidal behavior [49, 50]. Additionally, the late-onset attempters were less likely to meet diagnoses for any anxiety, any eating, and any PD than the early-onset attempters. These results suggest greater overall psychopathology among early-onset female attempters. Borderline and dependent PD, two PD that frequently cooccur [51] were also less likely to be diagnosed among late-onset female attempters when compared with their early-onset counterparts. This is coherent with our finding that older female attempters were less likely to have high scores of impulsivity and aggression, important factors in borderline PD. Higher levels of impulse-aggression have been associated with an earlier AAO of suicidal behavior [10, 52, 53]. Furthermore, diminished impulsivity has been reported in older patients with borderline PD [54]. Not surprisingly, older female attempters had a higher score on the premeditation subscale of the SIS scale than the younger subgroup, consistent with lower impulsivity scores among older female attempters. 

#### 4.1.3. Suicide-Related Measures and Their Correlates

 Total SIS mean score and the premeditation subscale were significantly higher among late-onset attempters compared to that of the younger attempters. Consistent with previous studies [55], our results suggest that late-onset attempters commit SA characterized by a higher intention and increased premeditation. Previous reports have suggested that older age and high suicide intent are risk factors for suicide completion among female attempters [56]. 

### 4.2. Men

 We found a trend for major depressive disorder to be more frequent in late-onset male attempters, whereas substance use, anxiety, and PD tended to be more likely among early-onset attempters. These results are congruent with data reported by Slama et al.[18] Furthermore, late-onset male attempters have lower odds of being discovered than early-onset attempters, which might suggest a greater intent of SA among older attempters. This is consistent with our finding of a trend for late-onset suicide attempters to use more violent methods such as drowning, hanging, or suffocation. Use of violent methods is a risk factor for completed suicide in men [56]. In addition, similarly to females, the late-onset male attempters were less likely to have high scores in the Brown Goodwin scale, measuring aggression. 

### 4.3. Limitations

 These study findings must be interpreted in light of several limitations. First, the cross-sectional design of this study limits the elucidation of the effects of impulsivity and aggression levels as well as of risk factors such as stressful life events over SA. Additionally, the retrospective assessment of these, especially the adverse childhood events, might have been subject to recall bias. Due to the length of the interview, we were unable to use a validated instrument. Future research should include valid measures such as the Childhood Trauma Questionnaire (CTQ) [12]. Finally, the small sample size of males might have affected our statistical power and explain the nonsignificance of at least some of our findings among men [15]. A larger male sample would be needed to confirm our preliminary results among males. 

## 5. Conclusion 

 Our study constitutes a major improvement in the understanding of the different subgroups of SA and support the existence of two subgroups defined by the AAO in both genders. Among females, late-onset of SA was associated with the presence of dysthymia and planning the SA ahead of time, while inversely associated with high levels of impulsivity, aggression, borderline PD, and dependent PD diagnoses when compared with their early-onset counterparts. Concerning males, late-onset male attempters were less likely to present with high aggression levels. Our study has important clinical implications and may contribute to planning more appropriate preventive and treatment regimens. Our results suggest that a younger person may need treatments directed to lowering impulsivity and aggressiveness after a first suicide attempt. In order to disentangle the heterogeneous nature of suicidal behavior and to improve current prevention measures, these findings require independent replication. 

## Figures and Tables

**Table 1:** Distribution of sociodemographic characteristics of individuals with a first SA by sex and age group.

	Women					Men				
Sociodemographic characteristics	Early onset (AAO 18–37)	Late onset (AAO ≥ 38)				Early onset (AAO 18–52)	Late onset (AAO ≥ 53)
	*N* = 91	*N* = 56				*N* = 64	*N* = 18
	%	%	OR	95% CI		%	%	OR	95% CI	
*Marital status*										
Single*	63.7	10.7	1	1	1	42.2	1.6	1	1	1
Married/cohabiting	26.3	66.0	1.49	5.56	39.91	35.9	61.1	12.91	1.54	107.72
Separated/widowed	9.9	23.2	1.39	4.22	46.14	21.9	33.3	11.57	1.26	105.82
*Years of education*										
≤8	20.9	51.8	**4.58**	**1.95**	**10.75**	34.4	11.1	1.35	.42	4.31
9 to 12	36.2	25.0	1.27	.52	3.09	31.3	50.0	.27	.05	1.46
>12*	42.8	23.2	1	1	1	32.8	38.8	1	1	1
*Socioeconomic status*										
Low-middle (1&2)	30.8	28.6	.76	.31	1.89	29.7	27.8	1.58	.26	9.48
Middle (3)	47.2	44.6	.77	.34	1.78	51.6	61.1	2.00	.39	10.36
Middle-high (4&5)*	22.0	26.8	1	1	1	18.8	11.1	1	1	1
*Urbanicity (when born)*										
Urban*	91.2	66.1	1	1	1	92.2	38.9	1	1	1
Rural	6.6	26.8	**5.61**	**2.02**	**15.60**	4.6	44.4	**22.48**	**4.81**	**104.94**
*Employment status*										
Unemployed (w/ and w/o subside)	30.8	16.1	.54	.22	1.29	28.1	66.7	2.29	.29	17.66
Employed*	57.1	55.4	1	1	1	60.9	11.1	1	1	1
Disability	8.8	16.1	2.22	.80	6.20	10.9	22.2	**7.43**	**1.51**	**36.6**
*Living arrangements*										
Alone	3.3	10.7	3.10	.74	12.98	12.5	16.7	1.28	.30	5.50
Family*	83.5	87.5	1	1	1	75.0	77.8	1	1	1
Nonfamily	13.2	1.8	1.29	.30	5.50	12.5	5.6	.49	.05	4.32
*Religious beliefs*										
Yes	61.5	78.6	**3.93**	**1.50**	**10.27**	37.5	27.8	1.69	.53	5.44
No*	33.0	10.7	1	1	1	53.1	66.7	1	1	1

*Reference group. **Significant results** with *P*-values <**.05** are indicated **in bold**.

**Table 2:** Current and lifetime risk factors for suicidal behavior among individuals with first SA by sex and age group.

	Women					Men				
Risk factors	Early onset (AAO 18–37)	Late onset (AAO ≥ 38)				Early onset (AAO 18–52)	Late onset (AAO ≥ 53)
	*N* = 91 (62%)	*N* = 56 (38%)				*N* = 64 (78%)	*N* = 18 (22%)
	%	%	OR	95% CI		%	%	OR	95% CI	
*Any childhood risk factor *	45.1	35.7	.68	.34	1.34	20.3	.0	NA	NA	NA
Physical abuse	12.5	9.1	.70	.23	2.14	10.9	.0	NA	NA	NA
Sexual abuse	8.0	7.3	.91	.25	3.26	3.1	.0	NA	NA	NA
Parental neglect	5.7	3.6	.62	.12	3.35	4.7	.0	NA	NA	NA
Emotional abuse	20.5	21.8	1.08	.48	2.47	7.8	.0	NA	NA	NA
*Adulthood risk factor*										
Physical abuse	20.5	18.2	.86	.36	2.04	1.6	.0	NA	NA	NA
Sexual abuse	14.8	3.6	.22	.05	1.00	.0	.0	NA	NA	NA
*Familial history of suicidal behavior*										
Family history of SA	16.9	22.4	1.42	.58	3.50	12.7	20.0	1.71	.38	7.63
Family history of Lethal SB	6.6	6.2	.95	.22	4.16	3.6	13.3	4.07	.52	31.72
*Stressful life events *										
Problems related to primary support group	76.4	81.5	1.36	.58	3.16	73.4	58.8	.517	.170	1.574
Social environment	6.9	14.8	2.35	.77	7.19	14.1	17.60	1.31	.313	5.485
School	8.0	.0	NA	NA	NA	7.8	.0	NA	NA	NA
Work	29.2	20.4	.62	.28	1.39	40.6	35.3	.80	.26	2.43
Housing	9.0	11.1	1.27	.41	3.87	7.8	5.9	.74	.08	6.78
Finances	5.6	16.7	**3.36**	**1.06**	**10.63**	23.4	17.6	.70	.18	2.77
Access to health care	13.5	16.7	1.28	.50	3.28	14.1	29.4	2.55	.72	8.97
Legal system	3.4	.0	NA	NA	NA	1.6	.0	NA	NA	NA
Other psychosocial problem	6.8	3.8	.55	.11	2.81	4.8	5.9	1.23	.12	12.63

**Table 3:** Clinical and Suicide attempt correlates of individuals with first SA by sex and age group.

	Women					Men				
Clinical and suicide correlates	Early onset (AAO 18–37)	Late onset (AAO ≥ 38)				Early onset (AAO 18–52)	Late onset (AAO ≥ 53)	
	%	%	OR	95% CI		%	%	OR	95% CI	
High impulsiveness	72.5	53.6	**.44**	**.22**	**.88**	65.6	55.6	.65	.23	1.89
High aggressiveness	53.8	33.9	**.44**	**.22**	**.88**	60.9	27.8	**.25**	**.08**	**.78**
No familial support	14.4	11.3	1.32	.47	3.72	29.5	22.2	1.46	.42	5.06
Agent used										
Ingestion/Laceration/Stabbing	97	93	1	1	1	82.8	83.3	1	1	1
Drowning/hanging/suffocation	1	0	NA	NA	NA	5.2	16.7	3.67	.67	20.05
Jumping/shooting	1	2	1.69	.10	27.63	3.5	.0	NA	NA	NA
Lesions/toxicity										
Mild*	83.3	73.4	1	1	1	77.9	64.5	1	1	1
Moderate	11.9	20.3	1.50	.55	4.10	16.3	25.8	1.94	.56	6.74
High	4.8	6.3	2.50	.53	11.73	5.8	9.7	8.54	.71	102.92
Reversibility										
Good*	95.2	92.2	1	1	1	94.3	87.1	1	1	1
Regular	4.0	7.8	1.29	.28	6.00	4.6	6.5	.87	.09	8.39
Bad	.8	.0	NA	NA	NA	1.1	3.2	1.06	.86	1.14
Odds of being discovered										
Low	7.1	10.9	2.41	.60	9.68	14.0	12.9	2.47	.34	17.83
Moderate	33.3	37.5	1.31	.63	2.73	47.7	58.1	**4.33**	**1.10**	**17.02**
High*	57.9	51.6	1	1	1	38.4	29.0	1	1	1
Interrupted attempt	55.1	45.1	.67	.37	1.34	47.5	52.5	1.42	.48	4.16
Intends repetition	15.0	18.2	1.26	.47	3.36	16.1	29.4	2.17	.61	7.70

	Mean	Mean	*T*-test	*P* value		Mean	Mean	*T*-test	*P* value	
SIS total score	13.63	28.08	−2.652	**.009**		19.70	22.06	−.305	.761	
Active preparation score	3.68	6.42	−1.818	.071		5.90	6.17	−.091	.928	
Premeditation score	9.96	21.66	−2.226	**.028**		13.80	15.89	−.295	.769	

*Reference group. **Significant results** with *P*-values <**.05** are indicated **in bold**.

## References

[1] Paykel ES, Myers JK, Lindenthal JJ, Tanner J (1974). Suicidal feelings in the general population: a prevalence study. *British Journal of Psychiatry*.

[2] Satcher D Remarks at the release of the surgeon general’s call to action to prevent suicide.

[3] Mann JJ (2003). Neurobiology of suicidal behaviour. *Nature Reviews Neuroscience*.

[4] Mann JJ, Brent DA, Arango V (2001). The neurobiology and genetics of suicide and attempted suicide: a focus on the serotonergic system. *Neuropsychopharmacology*.

[5] Anguelova M, Benkelfat C, Turecki G (2003). A systematic review of association studies investigating genes coding for serotonin receptors and the serotonin transporter—I. Affective disorders. *Molecular Psychiatry*.

[6] Arango V, Huang YY, Underwood MD, Mann JJ (2003). Genetics of the serotonergic system in suicidal behavior. *Journal of Psychiatric Research*.

[7] Oquendo MA, Baca-Garcia E, Mann JJ, Giner J (2008). Issues for DSM-V: suicidal behavior as a separate diagnosis on a separate axis. *American Journal of Psychiatry*.

[8] Nordstrom P, Samuelsson M, Asberg M (1995). Survival analysis of suicide risk after attempted suicide. *Acta Psychiatrica Scandinavica*.

[9] Beautrais AL (2003). Suicide and serious suicide attempts in youth: a multiple-group comparison study. *American Journal of Psychiatry*.

[10] Melhem NM, Brent DA, Ziegler M (2007). Familial pathways to early-onset suicidal behavior: familial and individual antecedents of suicidal behavior. *American Journal of Psychiatry*.

[11] Brodsky BS, Oquendo M, Ellis SP, Haas GL, Malone KM, Mann JJ (2001). The relationship of childhood abuse to impulsivity and suicidal behavior in adults with major depression. *American Journal of Psychiatry*.

[12] Roy A (2004). Relationship of childhood trauma to age of first suicide attempt and number of attempts in substance dependent patients. *Acta Psychiatrica Scandinavica*.

[13] Heikkinen ME, Isometsa ET, Aro HM, Sarna SJ, Lonnqvist JK (1995). Age-related variation in recent life events preceding suicide. *Journal of Nervous and Mental Disease*.

[14] Von Knorring AL, Bohman M, Von Knorring L, Oreland L (1985). Platelet MAO activity as a biological marker in subgroups of alcoholism. *Acta Psychiatrica Scandinavica*.

[15] Bellivier F, Golmard JL, Henry C, Leboyer M, Schürhoff F (2001). Admixture analysis of age at onset in bipolar I affective disorder. *Archives of General Psychiatry*.

[16] Schürhoff F, Golmard JL, Szoke A (2004). Admixture analysis of age at onset in schizophrenia. *Schizophrenia Research*.

[17] Kessler RC, Amminger GP, Aguilar-Gaxiola S, Alonso J, Lee S, Üstün TB (2007). Age of onset of mental disorders: a review of recent literature. *Current Opinion in Psychiatry*.

[18] Slama F, Courtet P, Golmard JL (2009). Admixture analysis of age at first suicide attempt. *Journal of Psychiatric Research*.

[19] Oquendo MA, Bongiovi-Garcia ME, Galfalvy H (2007). Sex differences in clinical predictors of suicidal acts after major depression: a prospective study. *American Journal of Psychiatry*.

[20] Dombrovski AY, Szanto K, Duberstein P, Conner KR, Houck PR, Conwell Y (2008). Sex differences in correlates of suicide attempt lethality in late life. *American Journal of Geriatric Psychiatry*.

[21] Lester D (1994). Sex differences in methods for suicide. *Perceptual and Motor Skills*.

[22] Zhang J, Mckeown RE, Hussey JR, Thompson SJ, Woods JR (2005). Gender differences in risk factors for attempted suicide among young adults: findings from the Third National Health and Nutrition Examination Survey. *Annals of Epidemiology*.

[23] Levinson D, Haklai Z, Stein N, Gordon ES (2006). Suicide attempts in Israel: age by gender analysis of a national emergency departments database. *Suicide and Life Threatening Behavior*.

[24] Nordentoft M, Branner J (2008). Gender differences in suicidal intent and choice of method among suicide attempters. *Crisis*.

[25] O’Carroll PW, Berman AL, Maris RW, Moscicki EK, Tanney BL, Silverman MM (1996). Beyond the tower of babel: a nomenclature for suicidology. *Suicide and Life Threatening Behavior*.

[26] Silverman MM, Berman AL, Sanddal ND, O’Carroll PW, Joiner TE (2007). Rebuilding the tower of babel: a revised nomenclature for the study of suicide and suicidal behaviors—part 1: background,rationale, and methodology. *Suicide and Life Threatening Behavior*.

[27] Sheehan DV, Lecrubier Y, Sheehan KH (1998). The Mini-International Neuropsychiatric Interview (M.I.N.I.): the development and validation of a structured diagnostic psychiatric interview for DSM-IV and ICD-10. *Journal of Clinical Psychiatry*.

[28] Egan V, Austin E, Elliot D, Patel D, Charlesworth P (2003). Personality traits, personality disorders and sensational interests in mentally disordered offenders. *Legal and Criminological Psychology*.

[29] Loranger A (1995). *International Personality Disorder Examination: ICD-10 Module*.

[30] Blasco-Fontecilla H, Baca-Garcia E, Dervic K (2009). Specific features of suicidal behavior in patients with narcissistic personality disorder. *Journal of Clinical Psychiatry*.

[31] Blasco-Fontecilla H, Baca-Garcia E, Duberstein P (2010). An exploratory study of the relationship between diverse life events and specific personality disorders in a sample of suicide attempters. *Journal of Personality Disorders*.

[32] Tyrer P (2009). Recurrence of self-harm & severity of personality disorder. *Acta Psychiatrica Scandinavica*.

[33] Baca-Garcia E, Parra PC, Perez-Rodriguez MM (2007). Psychosocial stressors may be strongly associated with suicide attempts. *Stress and Health*.

[34] Weissman MM, Leaf PJ, Livingston Bruce M, Florio L (1988). The epidemiology of dystmia in five communities: rates, risks, comorbidity, and treatment. *American Journal of Psychiatry*.

[35] Beck ASD, Herman I (1974). Development of suicidal intent scales. *The Prediction of Suicide*.

[36] Misson H, Mathieu F, Jollant F (2010). Factor analyses of the Suicidal Intent Scale (SIS) and the Risk-Rescue Rating Scale (RRRS): toward the identification of homogeneous subgroups of suicidal behaviors. *Journal of Affective Disorders*.

[37] Brown GL, Goodwin FK (1986). Cerebrosinal fluid correlates of suicide attempts and aggression. *Annals of the New York Academy of Sciences*.

[38] Oquendo MA, Baca-Garcia E, Graver R, Morales M, Montalvan V, Mann JJ (2001). Spanish adaptation of the Barratt Impulsiveness Scale (BIS-11). *European Journal of Psychiatry*.

[39] Baca-Garcia E, Oquendo MA, Saiz-Ruiz J, Mann JJ, De Leon J (2006). A pilot study on differences in aggression in New York City and Madrid, Spain, and their possible impact on suicidal behavior. *Journal of Clinical Psychiatry*.

[40] Zouk H, Tousignant M, Seguin M, Lesage A, Turecki G (2006). Characterization of impulsivity in suicide completers: clinical, behavioral and psychosocial dimensions. *Journal of Affective Disorders*.

[41] Weissman MM, Bland RC, Canino GJ (1999). Prevalence of suicide ideation and suicide attempts in nine countries. *Psychological Medicine*.

[42] Arensman E, Kerkhof AJ, Hengeveld MW, Mulder JD (1995). Medically treated suicide attempts: a four year monitoring study of the epidemiology in The Netherlands. *Journal of Epidemiology and Community Health*.

[43] Fekete S, Voros V, Osvath P (2005). Gender differences in suicide attempters in Hungary: retrospective epidemiological study. *Croatian Medical Journal*.

[44] Baca-Garcia E, Diaz-Sastre C, De Leon J, Saiz-Ruiz J (2000). The relationship between menstrual cycle phases and suicide attempts. *Psychosomatic Medicine*.

[45] Mann JJ, Waternaux C, Haas GL, Malone KM (1999). Toward a clinical model of suicidal behavior in psychiatric patients. *American Journal of Psychiatry*.

[46] Haw C, Hawton K (2008). Life problems and deliberate self-harm: associations with gender, age, suicidal intent and psychiatric and personality disorder. *Journal of Affective Disorders*.

[47] Duberstein PR, Conwell Y, Conner KR, Eberly S, Caine ED (2004). Suicide at 50 years of age and older: perceived physical illness, family discord and financial strain. *Psychological Medicine*.

[48] Murase S, Ochiai S, Ueyama M, Honjo S, Ohta T (2003). Psychiatric features of seriously life-threatening suicide attempters: a clinical study from a general hospital in Japan. *Journal of Psychosomatic Research*.

[49] Bernal M, Haro JM, Bernert S (2007). Risk factors for suicidality in Europe: results from the ESEMED study. *Journal of Affective Disorders*.

[50] Holmstrand C, Engstrom G, Traskman-Bendz L (2008). Disentangling dysthymia from major depressive disorder in suicide attempters’ suicidality, comorbidity and symptomatology. *Nordic Journal of Psychiatry*.

[51] Zanarini MC, Frankenburg FR, Vujanovic AA, Hennen J, Reich DB, Silk KR (2004). Axis II comorbidity of borderline personality disorder: description of 6-year course and prediction to time-to-remission. *Acta Psychiatrica Scandinavica*.

[52] Brent DA, Oquendo M, Birmaher B (2002). Familial pathways to early-onset suicide attempt: risk for suicidal behavior in offspring of mood-disordered suicide attempters. *Archives of General Psychiatry*.

[53] Brent DA, Oquendo M, Birmaher B (2003). Peripubertal suicide attempts in offspring of suicide attempters with siblings concordant for suicidal behavior. *American Journal of Psychiatry*.

[54] Stevenson J, Meares R, Comerford A (2003). Diminished impulsivity in older patients with borderline personality disorder. *American Journal of Psychiatry*.

[55] Caldera T, Herrera A, Kullgren G, Renberg ES (2007). Suicide intent among parasuicide patients in Nicaragua: a surveillance and follow-up study. *Archives of Suicide Research*.

[56] Skogman K, Alsen M, Ojehagen A (2004). Sex differences in risk factors for suicide after attempted suicide Sex differences in risk factors for suicide after attempted suicide-a follow-up study of 1052 suicide attempters. *Social Psychiatry and Psychiatric Epidemiology*.

